# Analysis of Risk Factors for Gastric Cancer and Precancerous Lesions: A Case–Control Study

**DOI:** 10.1111/1751-2980.13326

**Published:** 2025-01-26

**Authors:** Meng Jie Gao, Song Bo Li, Xiao Jing Zhu, Li Feng Zhang, Min Chen, Yong Quan Shi

**Affiliations:** ^1^ State Key Laboratory of Holistic Integrative Management of Gastrointestinal Cancers, National Clinical Research Center for Digestive Diseases Xijing Hospital, Air Force Medical University Xi'an, Shaanxi Province China; ^2^ Graduate Department, Xi'an Medical University Xi'an, Shaanxi Province China

**Keywords:** chronic gastritis, gastric neoplasms, gastric xanthoma, precancerous lesions

## Abstract

**Objectives:**

To investigate the correlation between gastric xanthoma (GX) and precancerous lesions (PCL) and gastric cancer (GC), and to explore other potential risk factors for PCL and GC in northwest China.

**Methods:**

A case–control study was conducted from September 2022 to September 2023 at Xijing Hospital, Air Force Medical University (Xi'an, Shaanxi Province, China). The patients who underwent gastroscopy were enrolled and divided into the chronic gastritis (CG) group (*n* = 423), PCL group (*n* = 422), and GC group (*n* = 415). The variables were selected through univariate analysis, including demographic information, dietary habits, lifestyle, gastroscopic findings, and *Helicobacter pylori* (*H. pylori*) infection. Multivariate logistic regression analysis was performed to analyze the factors associated with PCL and GC, and odds ratio (OR) and 95% confidence interval (CI) were calculated.

**Results:**

GX was more prevalent in the PCL group (14.93%) and the GC group (19.76%) than in the CG group (6.15%). Multivariate analysis revealed that age ≥ 50 years, male gender, rural residence, *H. pylori* infection, family history of GC, GX, and hypertension were independent risk factors for GC and PCL. Furthermore, a diet high in salt and spice, coupled with daily intake of less than 100 g of fresh fruits, might be associated with the occurrence of GC.

**Conclusion:**

Age ≥ 50 years, male gender, rural residence, family history of GC and *H. pylori* infection, presence of GX, and a history of hypertension may be risk factors for PCL and GC.

## Graphical abstract

A case–control study in northwest China demonstrated that age ≥ 50 years, male gender, rural residence, *Helicobacter pylori* (*H. pylori*) infection, family history of gastric cancer (GC), gastric xanthoma (GX), hypertension, diabetes mellitus, high‐salty/spicy food intake, low fresh fruit consumption, and gastric ulcers were risk factors for precancerous lesions (PCL) and GC. The detection rate of GX was significantly higher in the PCL and GC groups compared to the chronic gastritis (CG) group, highlighting its role as an independent risk factor for PCL and GC development. CAG, chronic atrophic gastritis; CNAG, chronic non‐atrophic gastritis; IEN, intraepithelial neoplasia; IM, intestinal metaplasia.

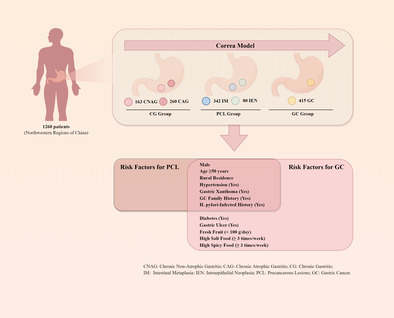

## 1 Introduction

Gastric cancer (GC) is one of the most prevalent malignant cancers in China, which accounts for 24.1% and 26.5% of the global incidence and death of GC in 2022, respectively [1]. Compared to its global distribution, GC also exhibits regional differences in China. The northwest region of China, including the Shaanxi Province, Gansu Province, Qinghai Province, and the Ningxia Hui Autonomous Region, and Xinjiang Uyghur Autonomous Region, has been reported to have a high incidence and mortality of GC. In 2016, GC ranked first in the incidence among all malignant tumors in both Gansu Province and Qinghai Province [2].

A multistage evolution of gastric mucosae occurs under continuous influence of various risk factors, ultimately leading to GC development. These risk factors encompass genetic predisposition, *Helicobacter pylori* (*H. pylori*) infection, lifestyle and dietary habits, as well as the underlying diseases of the individuals [3]. *H. pylori* is recognized as the primary risk factor for GC and has been classified as a class I carcinogen by the World Health Organization in 1994 [4]; however, only 17% of the individuals infected with *H. pylori* develop GC [5]. Eradication of the bacterium can only prevent against GC in up to 36% of the cases [6, 7, 8]. This indicates that, aside from *H. pylori* infection, there are other significant contributing factors to the development of GC. Therefore, it is crucial to investigate the risk factors for GC, particularly in high‐incidence areas.

Gastric xanthoma (GX), also known as “gastric lipid island,” is a lesion characterized by the deposition of foam cells. Previous studies have demonstrated a close association between GX and gastric mucosal lesions, such as mucosal atrophy, intestinal metaplasia (IM), intraepithelial neoplasia (IEN), and even GC. The prevalence of these lesions has been found to be significantly elevated in individuals with GX [9, 10, 11, 12, 13, 14]. Thus, we conducted a population‐based case–control study in northwest China, aiming to investigate the association between GX and GC, as well as that between GX and precancerous lesion (PCL), to identify the risk factors that might influence the development of these mucosal lesions, and to establish a scientific basis for implementing GC screening strategies in this region.

## 2 Patients and Methods

### 2.1 Ethical Approval

This study was approved by the Medical Ethics Committee of Xijing Hospital, Air Force Medical University (Xi'an, Shaanxi Province, China) (no. KY20232348‐C‐1) and was conducted in accordance with the Declaration of Helsinki (Brazil, 2013). Written informed consent was obtained from all patients before their enrollment. This study was registered on ClinicalTrials.gov (registration no. NCT06282484).

### 2.2 Patient Enrollment

This case–control study included 1260 patients who were admitted to the Department of Gastroenterology of our hospital between September 2022 and September 2023. The inclusion criteria were as follows: (a) participants aged 18–75 years who had resided in the northwest region of China (including the Shaanxi Province, Gansu Province, Qinghai Province, and Ningxia Hui Autonomous Region) for more than 10 years, regardless of their gender; (b) individuals who had undergone gastroscopy, as well as urea breath test (UBT) for *H. pylori* infection; and (c) those who consented to participate in the study. The exclusion criteria were: (a) patients aged < 18 years or > 75 years; (b) with a previous history of upper gastrointestinal (GI) surgery; (c) with a previous history of any malignant tumor; (d) inability to undergo gastroscopy because of conditions such as acute upper GI bleeding; (e) those who could not cooperate with the questionnaire survey due to mental disorders or other reasons; and (f) refusal to provide informed consent or having insufficient clinical data for analysis.

### 2.3 Patient Grouping

The gastroscopic images were reviewed by two endoscopists who were blinded to the patients’ characteristics. Narrow‐band imaging (NBI) was conducted for patients with IM, and gastric mucosal biopsy was performed in those with suspicious local lesions of GC or IEN. Diagnosis was made based on gastroscopic and/or histopathological findings. And the patients were then categorized into three groups: (a) the chronic gastritis (CG) group, including patients diagnosed with chronic atrophic gastritis (CAG) or chronic non‐atrophic gastritis (CNAG); (b) the PCL group, comprising those with IM or IEN; and (c) the GC group.

### 2.4 Data Collection and Sample Size Calculation

All patients were individually interviewed by rigorously trained researchers prior to the gastroscopic examination. Data of the patients were collected using a structured questionnaire consisting of 128 items across four categories. Gastroscopic and histopathological findings and UBT results were recorded in the electronic medical record system.

During the questionnaire survey, all participants were queried on the following items: (a) general demographic information, including age (years), height (meter), weight (kilograms), level of education, and long‐term residence (city or rural); (b) lifestyle and dietary habits, encompassing lifelong drinking habits (yes or no), lifelong smoking habits (yes or no), frequency of weekly intake of high salty food, overnight vegetables, processed food, and spicy food, coffee consumption (< 3 times/week or ≥ 3 times/week), and daily fresh fruit intake (< 100 g/day or ≥ 100 g/day); (c) medical history and prescribed medications for conditions such as hypertension, diabetes mellitus, coronary heart disease, and diseases of the biliary system, as well as the use of aspirin, metformin, and statins; and (d) family history of GC and previous *H. pylori* infection. Patients were required to answer the questionnaire based on their dietary habits over the past 5 years. If the patient had changed his or her dietary habits within the last 5 years, they were asked to provide their dietary habits within the past 2 years.

According to the principle that at least 10 outcome events (with PCL or GC) are needed for one variable in the regression analysis, a minimum sample size of 290 patients with PCL or GC was required.

### 2.5 Statistical Analysis

Statistical analyses were performed using SPSS Statistics version 26.0 (IBM, Armonk, NY, USA). Continuous variables with a normal distribution were presented as mean ± standard deviation, and the one‐way analysis of variance (ANOVA) was conducted for group comparisons. While those with non‐normal distribution were expressed as median and range, and the non‐parametric Mann–Whitney *U*‐test was performed for inter‐group comparisons. While categorical variables were presented as numbers and percentages or frequencies, the differences in which were compared between groups using the chi‐square test. Univariate analysis was first performed to identify the potential variables, and those with a *p* value of < 0.05 in the univariate analysis were further subjected into the multivariate analysis. Multivariate logistic regression analysis was employed to analyze the influencing factors of PCL and GC, and odds ratio (OR) and the corresponding confidence interval (CI) were calculated. A two‐sided *p* value of less than 0.05 was considered statistically significant.

## 3 Results

### 3.1 Demographic Characteristics of the Patients

**FIGURE 1:**
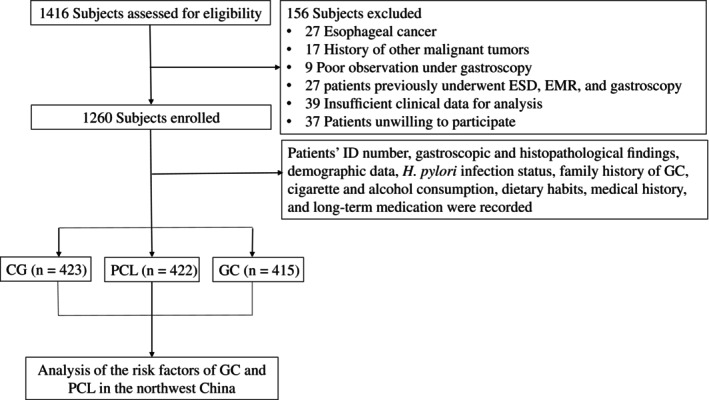
Flow diagram of patient enrollment. CG, chronic gastritis; EMR, endoscopic mucosal resection; ESD, endoscopic submucosal dissection; GC, gastric cancer; GX, gastric xanthoma; *H. pylori* , *Helicobacter pylori* ; ID, identification; PCL, precancerous lesion.

Figure 1 illustrates an overview of the process of patient enrollment. A total of 1260 patients were enrolled in the study. All patients came from the Shaanxi Province, Gansu Province, Qinghai Province, and the Ningxia Hui Autonomous Region, with the predominant ethnic group being Han population. Based on their final diagnosis, the patients were categorized into the CG group (*n* = 423), the PCL group (*n* = 422), and the GC group (*n* = 415), respectively. Compared with the CG group, there were more patients aged 50 years or elder, residing in rural areas, and with a lower level of education, as well as a male predominance, in the PCL and GC groups (all *p* < 0.001). However, there was no statistically significant difference in body mass index (BMI) between the CG and PCL or GC groups (both *p* > 0.05). The characteristics of the patients are summarized in Table 1.

**TABLE 1:** Demographic characteristics of the patients.

Variables	CG (*n* = 423)	PCL (*n* = 422)	*p* value*	GC (*n* = 415)	*p* value*
Location of the patients (*n*, %)			0.173		0.001
Shaanxi Province	280 (66.19)	301 (71.33)		248 (59.76)	
Gansu Province	84 (19.86)	70 (16.59)		83 (20.00)	
Qinghai Province	45 (10.64)	32 (7.58)		42 (10.12)	
Ningxia Hui Autonomous Region	14 (3.31)	19 (4.50)		42 (10.12)	
Ethnicity (*n*, %)			0.095		0.320
Han	416 (98.35)	420 (99.53)		404 (97.35)	
Others	7 (1.65)	2 (0.47)		11 (2.65)	
Age, years (mean ± SD)	49.16 ± 11.75	55.80 ± 8.89	< 0.001	60.35 ± 8.41	< 0.001
Age distribution (*n*, %)			< 0.001		< 0.001
< 50 years	189 (44.68)	94 (22.27)		38 (9.16)	
≥ 50 years	234 (55.32)	328 (77.73)		377 (90.84)	
Gender (*n*, %)			< 0.001		< 0.001
Male	194 (45.86)	263 (62.32)		312 (75.18)	
Female	229 (54.14)	159 (37.68)		103 (24.82)	
BMI, kg/m^2^ (mean ± SD)	23.01 ± 3.42	22.80 ± 3.06	0.331	23.41 ± 2.90	0.067
Education level (*n*, %)			< 0.001		< 0.001
University or above	145 (34.28)	98 (23.22)		82 (19.76)	
High school or below	278 (65.72)	324 (76.78)		333 (80.24)	
Residence (*n*, %)			< 0.001		< 0.001
City	316 (74.70)	255 (60.43)		184 (44.34)	
Rural	107 (25.30)	167 (39.57)		231 (55.66)	

Abbreviations: BMI, body mass index; CG, chronic gastritis; GC, gastric cancer; PCL, precancerous lesion; SD, standard deviation.

* Compared with the CG group.

### 3.2 Dietary Habits and Lifestyle

As shown in Table 2, smoking was significantly less common among patients in the PCL and GC groups than in the CG group (CG vs PCL: 76.60% vs 67.30%, *p* = 0.003; CG vs GC, 76.60% vs 63.86%, *p* < 0.001). Furthermore, individuals with GC showed higher rates of alcohol consumption, high intake of salty and spicy food, and a daily fresh fruit consumption of less than 100 g, when compared with the CG group. No significant differences were observed in other dietary habits and lifestyle across the three groups (all *p* > 0.05).

**TABLE 2:** Dietary habits and lifestyle of the patients.

Variables (*n*, %)	CG (*n* = 423)	PCL (*n* = 422)	*p* value*	GC (*n* = 415)	*p* value*
Smoking			0.003		< 0.001
No	99 (23.40)	138 (32.70)		150 (36.14)	
Yes	324 (76.60)	284 (67.30)		265 (63.86)	
Alcohol consumption			0.290		0.040
No	341 (80.61)	352 (83.41)		310 (74.70)	
Yes	82 (19.39)	70 (16.59)		105 (25.30)	
High‐salt food intake			0.202		< 0.001
+	295 (69.74)	311 (73.70)		225 (54.22)	
++	128 (30.26)	111 (26.30)		190 (45.78)	
High spicy food intake			0.317		< 0.001
+	307 (72.58)	319 (75.59)		232 (55.90)	
++	116 (27.42)	103 (24.41)		183 (44.10)	
Processed food intake			0.911		0.108
+	361 (85.34)	359 (85.07)		337 (81.20)	
++	62 (14.66)	63 (14.93)		78 (18.80)	
Overnight dish intake			0.090		0.055
+	259 (61.23)	282 (66.82)		227 (54.70)	
++	164 (38.77)	140 (33.18)		188 (45.30)	
Coffee consumption			0.837		0.869
+	411 (97.16)	411 (97.39)		404 (97.35)	
++	12 (2.84)	11 (2.61)		11 (2.65)	
Fresh fruit consumption			0.139		< 0.001
< 100 g/day	132 (31.21)	152 (36.02)		293 (70.60)	
≥ 100 g/day	291 (68.79)	270 (63.98)		122 (29.40)	

Abbreviations: CG, chronic gastritis; GC, gastric cancer; PCL, precancerous lesion; +, < 3 times/week; ++, ≥ 3 times/week.

* Compared with the CG group.

### 3.3 *H. pylori* Infection and Gastroscopic Findings

As shown in Table 3, in the GC and PCL groups, the proportion of patients with a history of *H. pylori* infection was significantly higher than that in the CG group (GC vs CG: 69.15% vs 55.79%, *p* < 0.001; PCL vs CG: 69.44% vs 55.79%, *p* < 0.001).

**TABLE 3:** *Helicobacter pylori* (*H. pylori*) infection and gastroscopic findings.

Variables (*n*, %)	CG (*n* = 423)	PCL (*n* = 422)	*p* value*	GC (*n* = 415)	*p* value*
History of *H. pylori* infection			< 0.001		< 0.001
No	187 (44.21)	129 (30.56)		128 (30.85)	
Yes	236 (55.79)	293 (69.44)		287 (69.15)	
GX			< 0.001		< 0.001
No	397 (93.85)	359 (85.07)		333 (80.24)	
Yes	26 (6.15)	63 (14.93)		82 (19.76)**	
Number of GX			0.450		0.513
Single	13 (50.00)	26 (41.27)		35 (42.68)	
Multiple	13 (50.00)	37 (58.73)		47 (57.32)	
Location of GX			0.735		0.420
Cardia or fundus	3 (11.54)	6 (9.52)		12 (14.63)	
Body	5 (19.23)	17 (26.98)		25 (30.49)	
Antrum	18 (69.23)	40 (63.49)		45 (54.88)	
Fundic gland polyp			0.388		0.048
No	372 (87.94)	379 (89.81)		382 (92.05)	
Yes	51 (12.06)	43 (10.19)		33 (7.95)	
Gastric ulcer			0.610		< 0.001
No	416 (98.35)	413 (97.87)		376 (90.60)	
Yes	7 (1.65)	9 (2.13)		39 (9.40)	
Duodenal ulcer			0.123		0.133
No	409 (96.69)	415 (98.34)		408 (98.31)	
Yes	14 (3.31)	7 (1.66)		7 (1.69)	
Gastroesophageal reflux disease			0.331		0.892
No	342 (80.85)	352 (83.41)		334 (80.48)	
Yes	81 (19.15)	70 (16.59)		81 (19.52)	

Abbreviations: CG, chronic gastritis; GC, gastric cancer; GX, gastric xanthoma; PCL, precancerous lesion.

* Compared with the CG group.

** *p* = 0.065 for GC group vs PCL group.

**FIGURE 2:**
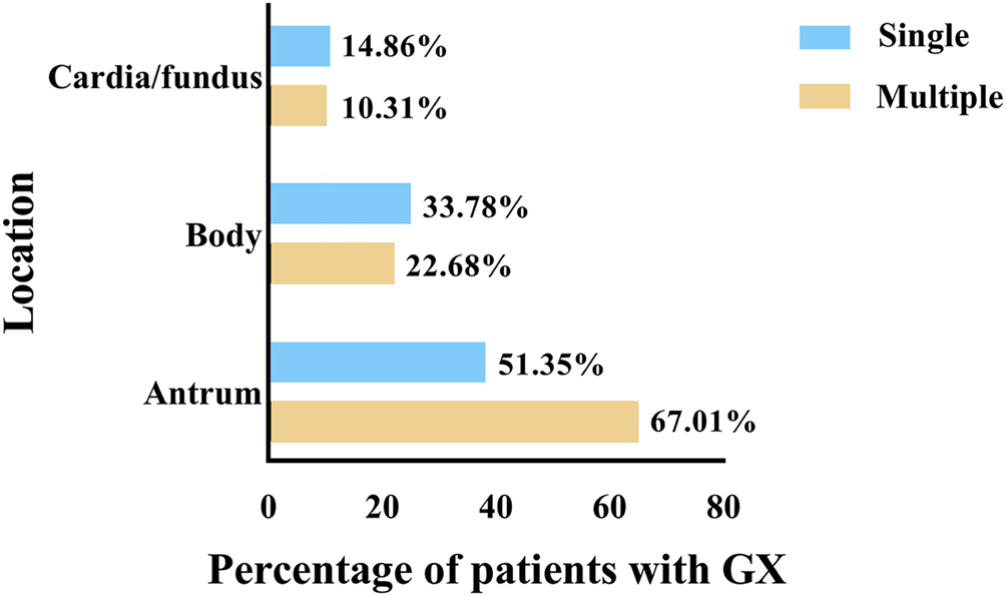
Distribution of gastric xanthoma (GX) in the stomach. For multiple GX, the location of the lesion of the largest diameter is considered the main site of occurrence.

Among the 1260 patients, GX was detected in 171 patients, resulted in a detection rate of 13.57%. Of these, 97 (56.73%) patients had multiple GX and 74 (43.27%) patients had single GX, respectively. The detection rate of GX gradually increased from the CG group (6.15%) to both the PCL group (14.93%) and the GC group (19.76%). Though the GC group had a higher prevalence of GX than the PCL group, the difference did not reach statistical significance (*p* = 0.065). We further divided CG into CNAG and CAG, and PCL into IM and IEN. Thus, all patients were categorized into five groups according to the Correa cascade (Table 4). Interestingly, the detection rate of GX significantly increased alongside the Correa cascade (*p* < 0.001). However, there were no statistically significant differences in the types and locations of GX among the CG, PCL, and GC groups (Table 3, Figure 2); the predominant site of GX was the gastric antrum, regardless of whether they appeared as single or multiple lesions (Table S1). Notably, among the 82 GC patients with GX, 31 had lesions located at the same site. Among them, GX in 3 cases were found at the cardia or fundus, 12 cases at the antrum, and 16 cases at the body of the stomach (Table 5). These results indicated that the coexistence rate of the lesions in the body of the stomach (13.68%) was significantly higher than that in the cardia or fundus (2.70%) and the antrum (6.42%) (*p* = 0.005). Among the three groups, the detection rate of gastric fundic gland polyp was the lowest, whereas that of gastric ulcer was the highest.

**TABLE 4:** The detection rate of gastric xanthoma (GX) in patients with different gastric conditions.

GX (*n*, %)	CNAG (*n* = 163)	CAG (*n* = 260)	IM (*n* = 342)	IEN (*n* = 80)	GC (*n* = 415)	*p* value
No	158 (96.93)	239 (91.92)	291 (85.09)	68 (85.00)	333 (80.24)	< 0.001
Yes	5 (3.07)	21 (8.08)	51 (14.91)	12 (15.00)	82 (19.76)	

Abbreviations: CAG, chronic atrophic gastritis; CNAG, chronic non‐atrophic gastritis; GC, gastric cancer; IEN, intraepithelial neoplasia; IM, intestinal metaplasia.

**TABLE 5:** Detection of gastric cancer (GC) and gastric xanthoma (GX) at the same location.

Location of GC	GX (*n*)		Total (*n*)	Coexistence rate (%)	*p* value
	Yes	No			
Cardia or fundus	3	108	111	2.70	0.005
Body	16	101	117	13.68	
Antrum	12	175	187	6.42	

### 3.4 Underlying Diseases and Long‐Term Medication

As shown in Table 6, in both the GC and PCL groups, the proportion of patients with at least one first‐degree relative affected by GC was significantly higher compared to the CG group (GC vs CG: 10.84% vs 2.60%, *p* < 0.001; PCL vs CG: 14.93% vs 2.60%, *p* < 0.001). Among the three groups, the GC group had the highest proportion of patients with hypertension, diabetes mellitus, and those who had been taking metformin for more than one year.

**TABLE 6:** Underlying diseases and long‐term medication.

Variables (*n*, %)	CG (*n* = 423)	PCL (*n* = 422)	*p* value*	GC (*n* = 415)	*p* value*
Family history of GC			< 0.001		< 0.001
No	412 (97.40)	359 (85.07)		370 (89.16)	
Yes	11 (2.60)	63 (14.93)		45 (10.84)	
Hypertension			< 0.001		< 0.001
No	385 (91.02)	350 (82.94)		322 (77.59)	
Yes	38 (8.98)	72 (17.06)		93 (22.41)	
Diabetes mellitus			0.834		< 0.001
No	411 (97.16)	409 (96.92)		376 (90.60)	
Yes	12 (2.84)	13 (3.08)		39 (9.40)	
Coronary heart disease			0.050		0.169
No	412 (97.40)	400 (94.79)		397 (95.66)	
Yes	11 (2.60)	22 (5.21)		18 (4.34)	
Gallbladder diseases			0.243		0.123
No	334 (78.96)	319 (75.59)		345 (83.13)	
Yes	89 (21.04)	103 (24.41)		70 (16.87)	
Aspirin use			0.119		0.256
No	410 (96.93)	400 (94.79)		396 (95.42)	
Yes	13 (3.07)	22 (5.21)		19 (4.58)	
Metformin use			0.363		0.008
No	415 (98.11)	410 (97.16)		393 (94.70)	
Yes	8 (1.89)	12 (2.84)		22 (5.30)	
Statins use			0.290		0.345
No	409 (96.69)	402 (95.26)		396 (95.42)	
Yes	14 (3.31)	20 (4.74)		19 (4.58)	

*Note*: Patients who have taken medication for more than one year are considered to be on long‐term medication.

Abbreviations: CG, chronic gastritis; GC, gastric cancer; GX, gastric xanthoma; PCL, precancerous lesion.

* Compared with the CG group.

### 3.5 Multivariate Analysis of Risk Factors for PCL and GC 

Using the CG group as a reference, variables with a *p* value of less than 0.05 were selected for inclusion in the multivariate conditional logistic regression analysis of GC and PCL (Table 7). Age of 50 years or elder, male gender, rural residence, history of *H. pylori* infection, and a family history of GC, as well as GX and hypertension, were identified as independent risk factors for GC and PCL. In addition, other risk factors for GC included the consumption of high salty or spicy food at least three times per week, consuming less than 100 g of fresh fruit per day, and the presence of gastric ulcers and diabetes mellitus. None of the other variables showed a statistically significant influence on GC and PCL (*p* > 0.05). We employed the CNAG and CAG patients as the control groups, sequentially conducting the univariate and multivariate analyses. The results obtained were largely consistent with the previously stated conclusions (Tables 7, S2, and S3).

**TABLE 7:** Multivariate analysis of risk factors associated with gastric precancerous lesion (PCL) and gastric cancer (GC).

Variables	PCL (*n* = 422)			GC (*n* = 415)		
	OR	95% CI	*p* value	OR	95% CI	*p* value
Age (≥ 50 years vs < 50 years)	2.483	1.790–3.445	< 0.001	6.509	3.952–10.721	< 0.001
Gender (male vs female)	1.961	1.453–2.646	< 0.001	3.003	2.046–4.406	< 0.001
Residence (rural vs city)	1.933	1.400–2.669	< 0.001	2.885	1.967–4.231	< 0.001
History of *H. pylori* infection (yes vs no)	2.036	1.487–2.788	< 0.001	2.103	1.431–3.091	< 0.001
GX (yes vs no)	2.174	1.307–3.616	0.003	2.640	1.486–4.690	0.001
Gastric ulcer (yes vs no)	1.247	0.432–3.603	0.683	5.581	2.044–15.238	0.001
High‐salt food intake (++ vs +)	1.330	0.950–1.863	0.097	2.219	1.505–3.273	< 0.001
High spicy food intake (++ vs +)	1.550	0.773–1.550	0.610	2.552	1.712–3.803	< 0.001
Fresh fruit consumption (< 100 g/day vs ≥ 100 g/day)	0.845	0.613–1.164	0.303	5.552	3.787–8.141	< 0.001
Family history of GC (yes vs no)	6.072	3.063–12.037	< 0.001	5.220	2.227–11.966	< 0.001
Hypertension (yes vs no)	1.635	1.037–2.579	0.034	2.120	1.243–3.616	0.006
Diabetes mellitus (yes vs no)	1.283	0.556–2.963	0.559	3.112	1.387–6.982	0.006

*Note*: The chronic gastritis group was used as the reference.

Abbreviations: CI, confidence interval; GX, gastric xanthoma; *H. pylori* , *Helicobacter pylori* ; OR, odds ratio; +, < 3 times/week; ++, ≥ 3 times/week.

## 4 Discussion

In this study we found that age, gender, residence, *H. pylori* infection, family history of GC, presence of GX, and underlying diseases were associated with GC and PCL. In addition, several dietary habits were also found to be significantly associated with GC.

Previous studies have demonstrated a correlation between age or gender and GC as well as PCL [3, 15]. According to data from the China Cancer Center, the 2016 crude incidence rate of GC for men was approximately 2.2 times of that for women (39.02/100 000 vs 17.82/100 000) [2]. Its incidence among Chinese residents is relatively low before the age of 40 years, which increases sharply thereafter, reaching its peak among those aged 80 years and above regardless of their gender. Similar to the incidence pattern of GC, the detection rate of PCL in male Chinese residents surpasses that of their female counterparts. Furthermore, the detection rate of PCL increases with age [16, 17]. This phenomenon may be attributed to the diminished pain sensitivity and the absence of typical GI symptoms as individuals age [18].

In our study, the distribution of education level and residence among patients with GC and PCL showed discernible disparities. Compared with the CG group, significantly higher proportions of patients in the GC and PCL groups had a lower education level and resided in rural areas, which is consistent with findings from a national epidemiological report in 2016 [2]. Moreover, the multivariate analysis indicated that residing in rural area was an independent risk factor for GC and PCL. This might be due to the reason that medical and healthcare resources are not as good as those in the city, which results in inadequate access to endoscopic monitoring and *H. pylori* testing. Furthermore, the prolonged consumption of well water, surface water, and other easily contaminated sources was strongly associated with the incidence of GC [19].

*H. pylori* is considered a significant risk factor for the progression of gastric mucosal lesions and has been classified as a class I carcinogen by the International Agency for Research on Cancer since 1994. The results of a case–control study conducted in China suggested that 78.5% of non‐cardia GC and 62.1% of cardia cancer cases could be attributed to *H. pylori* infection [20]. Additionally, a study in Korea showed that *H. pylori* was associated with PCL (OR 1.40, 95% CI 1.29–1.50, *p* < 0.001), and such risk was significantly increased in individuals under 40 years of age (OR 3.65, 95% CI 0.42–31.67, *p* < 0.001) [21]. In the current study, the prevalence of *H. pylori* infection in the CG group was comparable to that of the general population in northwest China (55.79% vs 51.8%) [22]. However, the *H. pylori* infection rate in our study was 69.15% in the GC group and 69.44% in the PCL group, which is consistent with previous studies [23], indicating a higher prevalence of *H. pylori* infection among patients with GC and PCL.

A family history of at least one first‐degree relative with GC is also a potential risk factor for both GC and PCL, as indicated by an OR of 5.220 and 6.072, respectively. Our results are consistent with a previous study that the familial aggregation of PCL and GC might have been due to genetic susceptibility and exposure to the same environmental factors such as the strong toxicity of *H. pylori* infection (if any) and common dietary habits and patterns within a family [17]. A previous cohort study also showed that individuals with a family history of PCL and GC had 2.5 and 3.8 times higher risk of developing non‐cardia GC compared to those with relatives showing mild gastric mucosal lesions [24]. Recent studies have shown that individuals with a familial predisposition to GC were at an elevated risk for developing IM and IEN. Furthermore, these patients showed an accelerated progression from PCL to GC [25, 26]. These findings underscore the significance of a family history of GC as a crucial risk factor for the development of PCL and GC. Therefore, when assessing the risks of GC and PCL, physicians should prioritize the evaluation of their family history of GC.

Furthermore, specific dietary habits have been associated with the development of GC. Multivariate conditional logistic regression analysis indicated that frequent intake of high‐salt and spicy food, as well as daily fresh fruit intake < 100 g, were risk factors for GC. In the high‐risk region for GC in northwest China, residents often rely on pickled vegetables as their primary source of winter vegetables. Salted, pickled, and smoked food commonly contain elevated levels of nitrate and nitrite, which can lead to the formation of carcinogenic *N*‐nitroso compounds (NOCs) upon consumption. This contributes to the onset of GC [27]. Consumption of fruits and vegetables rich in vitamin C, particularly citrus fruits, can mitigate oxidative damage to gastric mucosa and inhibit the production of NOCs, thereby lowering the likelihood of GC [28]. A 10‐year cohort study conducted in China showed that diets high in salt and spicy food were associated with an increased risk of GC, independent of patient's gender [29]. However, a study encompassing 564 748 patients found that individuals who regularly consumed spicy food might experience a 12% decrease in all‐cause mortality risk [30]. The difference in correlation between spicy food and GC may be related to different dietary capacity and frequency, populations, regions, and environmental factors.

Although GX was defined as a benign lesion, it has been reported to correlate with multiple stages during the development of GC. Yi et al [31] and Sekikawa et al [32] reported a significantly higher detection rate of CAG in the GX group than that in the non‐GX groups. The presence of GX often indicates more severe and extensive gastric mucosal atrophy [14, 31, 32]. A cross‐sectional study revealed that gastric IM and IEN were detected more frequently in the GX group than in the non‐GX group [12]. A binary logistic regression analysis demonstrated that IM was a statistically significant independent risk factor of GX [13]. GX was detected in 5.44%–40.4% of GC [11, 14] and in 41.67%–72.5% of early gastric cancer (EGC) [10, 32, 33, 34, 35, 36]. Studies have indicated that GX is an independent risk factor for the development of GC under various mucosal backgrounds, including those with or without *H. pylori* infection [34, 36], EGC, and metachronous or synchronous GC following endoscopic treatment of EGC [33, 37]. Moreover, a previous study with a median follow‐up period of 63 months, involving 1823 patients who underwent physical examinations, revealed that the incidence of EGC was significantly higher in the group with GX compared to that without GX (14.0% vs 0.80%, *p* < 0.001) [32]. In summary, there is a significant link between GX and GC. GX may play an important role in the onset and progression of GC through the following pathways. First, the presence of GX leads to an increased activation and release of oxygen free radicals [38]. These oxygen free radicals, through their specific mechanisms of action, cause DNA damage and are implicated in the development of various malignancies. Second, the presence of GX reflects the severity and disease duration of CG [35]. Third, molecular alterations occur in the gastric mucosa where GX forms, and these abnormal molecular changes (such as microsatellite instability [MSI] or methylation of tumor suppressor genes) do not easily normalize following the eradication of *H. pylori* . This explains why GX can still be observed in the gastric mucosa after *H. pylori* eradication [37].

Previous studies have shown that GX frequently appears in a single form at the gastric antrum [12, 31]. The incidence of GX was reported as 0.02%–0.38% in Western countries [39, 40], 0.23%–5.70% in Turkey [41], and 0.78%–12.00% in Eastern Asian countries (China [12, 13, 14], Japan [10, 42], and Korea [31]). The total detection rate of GX in our study was 13.57% (171/1260), which is slightly higher than those in previous studies. The major location of GX was the gastric antrum—either single or multiple—which was consistent with previously reported. However, multiple‐type GX was more common than single‐type GX in this study. Sekikawa et al [10] reported that GX of the cardia and fundus was closely related to the occurrence of GC. While in our study, the coexistence rate of GX in the body of the stomach with GX was significantly higher than that in other parts (body vs cardia/fundus vs antrum: 13.68% vs 2.70% vs 6.42%, *p* = 0.005). These differences might reflect the ethnic and geographical factors. Interestingly, the prevalence of GX in the PCL group (14.93%) and GC group (19.76%) was significantly higher than that in the CG group (6.15%). It was also found that the detection rate of GX significantly increased alongside the Correa cascade (from CNAG to CAG, IM, IEN, and GC). When using patients in the CG, CNAG, or CAG groups as the control population, multivariate analysis consistently showed that GX served as an independent risk factor for GC and PCL (Tables 7, S2, and S3). These results indicate a strong correlation between GX and the development of GC and PCL.

There is a scarcity of conclusively established research on the etiology and pathogenesis of GX. As a key risk factor for the occurrence and development of GC, the correlation between *H. pylori* and GX has also sparked researchers' interest. In this study, *H. pylori* infection rate in the GX group was comparable to that in the non‐GX group (66.08% vs 64.55%, *p* = 0.698; Table S4), aligning with previous studies [31, 36, 41]. Meanwhile, some studies have suggested that GX serves as a clear marker of *H. pylori* infection, which can objectively reflect the degree of gastric mucosal injury even after *H. pylori* eradication [33, 34, 42]. Scholars hypothesize that some GX may arise as a result of phagocytosis of *H. pylori* by macrophages and monocytes. Even after the eradication of *H. pylori* , these already transformed cells may continue to exist and form a component of GX [43].

Currently, the general consensus is that a hyperglycemic state promotes the excessive generation of oxygen radicals, thereby creating favorable conditions for the formation of GX. A large‐scale study found that elevated fasting blood glucose (OR 3.347, 95% CI 1.170–9.575, *p* < 0.05) was an independent risk factor for the development of GX [12]. Meanwhile, another study found that GX patients had significantly higher glycosylated hemoglobin levels (5.88% ± 0.07% vs 5.68% ± 0.02%, *p* = 0.0003) and higher incidence of diabetes mellitus compared to non‐GX individuals and that the incidence of diabetes mellitus in GX patients was significantly higher than that in non‐GX patients (22.4% vs 9.1%, *p* < 0.0001) [32]. In our study, there was no significant difference in the proportion of diabetes mellitus between the two groups (GX vs non‐GX: 5.26% vs 5.05%, *p* = 0.906; Table S4), which might be attributed to the fact that the diabetic population in this region place a greater emphasis on blood glucose control. It is noteworthy that the formation of GX is not only related to abnormal glucose metabolism but also influenced by multiple factors such as chronic inflammation of gastric mucosa and oxidative stress. These pathophysiological processes may also damage the blood vessel walls, leading to wall thickening, luminal narrowing and, consequently, an increased risk of hypertension. Despite our GX group having a higher prevalence rate of hypertension compared to the non‐GX group, the difference was not statistically significant (20.47% vs 15.43%, *p* = 0.096; Table S4), thereby necessitating further in‐depth studies to confirm the specific correlation between GX and hypertension.

This study had several advantages. First, over the past two to three decades, there has been a declining trend in the incidence of GC in China. However, the incidence and mortality of GC remain high in the northwest China. Therefore, it is necessary to investigate the risk factors associated with GC and PCL in this region and compare them with those in other regions of China. Second, all baseline characteristics of the patients were collected by professional researchers using standardized questionnaires, and the quality of data collection was strictly controlled to ensure the accuracy of the results. Third, this is one of the few studies that systematically explored the relationship between GX, GC, and PCL while identifying GX as an independent variable.

Nevertheless, there were some limitations to this study. First, as a retrospective study, despite the implementation of rigorous quality control measures, accurately determined, self‐reported intake of high‐salt, spicy food and fresh fruits remained challenging. Consequently, it was difficult to completely avoid recall bias and misclassification bias. Second, not all patients with CAG and IM underwent endoscopic biopsies, suggesting that the CG group might have included some patients with IM. However, this might not have changed the ultimate outcome of the study. Third, the present study had no follow‐up data of the recruited patients, and hence more prospective cohort studies with follow‐up are warranted to further determine the relationship between GX and GC.

In conclusion, we found a higher detection rate of GX in both PCL and GC than CG and demonstrated that the presence of GX was an independent risk factor for the development of GC and PCL. Therefore, careful screening for gastric neoplastic lesions is required for patients with GX during the endoscopic examination. Age of 50 years or elder, male gender, rural residence, *H. pylori* infection, family history of GC, and hypertension may be independent risk factors for GC and PCL. In addition, a diet high in salt and spicy food, intake of fresh fruits of < 100 g/day, gastric ulcer, and diabetes mellitus may be related to GC development.

## Conflicts of Interest

The authors declare no conflicts of interest.

## Supporting information


**Supplementary Table S1.** Distribution of gastric xanthoma (GX).
**Supplementary Table S2.** Multivariate analysis of risk factors associated with gastric precancerous lesion (PCL) and gastric cancer (GC).
**Supplementary Table S3.** Multivariate analysis of risk factors associated with gastric precancerous lesion (PCL) and gastric cancer (GC).
**Supplementary Table S4.** Comparison of *Helicobacter pylori* (*H. pylori*) infection history, diabetes mellitus, and hypertension between gastric xanthoma (GX) group and non‐GX group.
