# Supplementary material for: Analysis of Risk Factors for Gastric Cancer and Precancerous Lesions: A Case–Control Study
Source: J Dig Dis. 2025 Jan 26;25(11-12):674–84. doi: 10.1111/1751-2980.13326 (PMC11877994; doi:10.1111/1751-2980.13326)
Supplement: Supplementary file 1 — Supplementary Table S1. Distribution of gastric xanthoma (GX). Supplementary Table S2. Multivariate analysis of risk factors associated with gastric precancerous lesion (PCL) and gastric cancer (GC). Supplementary Table S3. Multivariate analysis of risk factors associated with gastric precancerous lesion (PCL) and gastric cancer (GC). Supplementary Table S4. Comparison of Helicobacter pylori (H. pylori) infection history, diabetes mellitus, and hypertension between gastric xanthoma (GX) group and non‐GX group. [file CDD-25-674-s001.docx]

Supplementary Table 1. Distribution of gastric xanthoma (GX).

| Characteristics (n, %) | Cardia or fundus | Body | Antrum |
| --- | --- | --- | --- |
| Number of GX |  |  |  |
| Single (n = 74) | 11 (14.86) | 25 (33.78) | 38 (51.35) |
| Multiple (n = 97) | 10 (10.31) | 22 (22.68) | 65 (67.01) |

The location of the lesion of the largest diameter is considered the main site of occurrence.

Supplementary Table 2. Multivariate analysis of risk factors associated with gastric precancerous lesion (PCL) and gastric cancer (GC).

| Variables | PCL (n = 422) | | | GC (n = 415) | | | |
| --- | --- | --- | --- | --- | --- | --- | --- |
|  | OR | 95% CI | *p* value | OR | 95% CI | | *p* value |
| Age (≥50 years vs <50 years) | 5.746 | 3.618–9.126 | <0.001 | 19.947 | 10.196–39.022 | | <0.001 |
| Gender (male vs female) | 2.889 | 1.846–4.521 | <0.001 | 4.300 | 2.400–7.705 | | <0.001 |
| Residence (rural vs city) | 1.804 | 1.073–3.035 | 0.026 | 2.938 | 1.608–5.369 | | <0.001 |
| Education level (low vs high) | 1.821 | 1.094–3.030 | 0.021 | — | — | | — |
| History of *H. pylori* infection (yes vs no) | 3.540 | 2.230–5.618 | <0.001 | 4.654 | 2.526–8.572 | | <0.001 |
| GX (yes vs no) | 3.764 | 1.369–10.344 | 0.010 | 5.193 | 1.695–15.909 | | 0.004 |
| Gastric ulcer (yes vs no) | 1.634 | 0.301–8.859 | 0.569 | 5.581 | 2.044–15.238 | | 0.001 |
| High salt food intake (++ vs +) | 1.319 | 0.789–2.205 | 0.291 | 2.304 | 1.261–4.210 | | 0.007 |
| High spicy food intake (++ vs +) | 1.124 | 0.669–1.886 | 0.659 | 2.242 | 1.231–4.083 | | 0.008 |
| Fresh fruit intake (<100 g/day vs ≥100 g/day) | 0.681 | 0.415–1.119 | 0.130 | 7.148 | 3.960–12.900 | | <0.001 |
| Family history of GC (yes vs no) | 5.885 | 1.917–18.063 | 0.002 | — | — | | — |
| Hypertension (yes vs no) | 2.230 | 1.007–4.937 | 0.048 | 2.931 | 1.262–6.807 | 0.012 | |

*Note:* The patients with chronic non-atrophic gastritis were regarded as the reference.

Low education level indicates high school or below, and high education level indicates university or above.

Abbreviations: CI, confidence interval; GX, gastric xanthoma; *H. pylori*, *Helicobacter pylori*; OR, odds ratio; +, <3 times/week; ++, ≥3 times/week.

Supplementary Table 3. Multivariate analysis of risk factors associated with gastric precancerous lesion (PCL) and gastric cancer (GC).

| Characteristics | PCL (n = 422) | | | GC (n = 415) | | |
| --- | --- | --- | --- | --- | --- | --- |
|  | OR | 95% CI | *p* value | OR | 95% CI | *p* value |
| Age (≥50 years vs <50 years) | 1.541 | 1.065–2.231 | 0.022 | 3.812 | 2.206–6.590 | <0.001 |
| Gender (male vs female) | 1.754 | 1.261–2.440 | 0.001 | 2.780 | 1.842–4.196 | <0.001 |
| Residence (rural vs city) | 1.832 | 1.285–2.612 | 0.001 | 2.929 | 1.941–4.421 | <0.001 |
| History of *H. pylori* infection (yes vs no) | 1.502 | 1.061–2.126 | 0.022 | 1.547 | 1.017–2.355 | 0.042 |
| GX (yes vs no) | 1.792 | 1.042–3.080 | 0.035 | 2.333 | 1.271–4.283 | 0.006 |
| Gastric ulcer (yes vs no) | 1.015 | 0.320–3.219 | 0.980 | 5.558 | 1.835–16.837 | 0.002 |
| High salt food intake (++ vs +) | 0.736 | 0.506–1.069 | 0.108 | 2.099 | 1.385–3.181 | <0.001 |
| High spicy food intake (++ vs +) | 1.040 | 0.698–1.548 | 0.847 | 2.820 | 1.820–4.369 | <0.001 |
| Fresh fruit intake (<100 g/day vs ≥100 g/day) | 1.049 | 0.730–1.507 | 0.798 | 5.085 | 3.376–7.661 | <0.001 |
| Family history of GC (yes vs no) | 6.434 | 2.852–14.505 | <0.001 | 5.337 | 2.096–13.591 | <0.001 |
| Hypertension (yes vs no) | 1.593 | 0.962–2.637 | 0.070 | 2.075 | 1.160–3.711 | 0.014 |
| Diabetes mellitus (yes vs no) | 0.659 | 0.260–1.667 | 0.378 | 3.161 | 1.308–7.643 | 0.011 |

*Note:* The patients with chronic atrophic gastritis were regarded as the reference.

Abbreviations: CI, confidence interval; GX, gastric xanthoma; *H. pylori*, *Helicobacter pylori*; OR, odds ratio; +, <3 times/week; ++, ≥3 times/week.

Supplementary Table 4. Comparison of *Helicobacter pylori* (*H. pylori*) infection history, diabetes mellitus, and hypertension between gastric xanthoma (GX) group and non-GX group.

| Characteristics (n, %) | GX group (n = 171) | Non-GX group (n = 1089) | *p* value |
| --- | --- | --- | --- |
| History of *H. pylori* infection |  |  | 0.698 |
| Yes | 113 (66.08) | 703 (64.55) |  |
| No | 58 (33.92) | 386 (35.45) |  |
| Diabetes mellitus |  |  | 0.906 |
| Yes | 9 (5.26) | 55 (5.05) |  |
| No | 162 (94.74) | 1034 (94.95) |  |
| Hypertension |  |  | 0.096 |
| Yes | 35 (20.47) | 168 (15.43) |  |
| No | 136 (79.53) | 921 (84.57) |  |
